# A case of esophagojejunal varices rupture after proximal gastrectomy with double-tract reconstruction

**DOI:** 10.1186/s40792-020-0775-6

**Published:** 2020-01-09

**Authors:** Naoki Shinno, Ryohei Kawabata, Haruna Furukawa, Seiichi Goda, Toshinori Sueda, Tae Matsumura, Chikato Koga, Shingo Noura, Junzo Shimizu, Atsuya Okada, Junichi Hasegawa

**Affiliations:** 1 Departments of Surgery, Osaka International Cancer Institute, 3-1-69 Otemae, Chuo-ku, Osaka, 541-8567 Japan; 2 Departments of Surgery, Osaka Rosai Hospital, 1179-3 Nagasone-cho, Kita-ku, Sakai, 591-8025 Japan; 3 Departments of Radiology, Osaka Rosai Hospital, 1179-3 Nagasone-cho, Kita-ku, Sakai, 591-8025 Japan; 4 Departments of Surgery, Toyonaka Municipal Hospital, 4-14-1 Shibahara-cho, Toyonaka, 560-8565 Japan

**Keywords:** PTO, Esophagojejunal varices, Proximal gastrectomy

## Abstract

**Background:**

The varices after proximal or total gastrectomy are uncommon because the supplying vessels are all divided. Emergent upper gastrointestinal endoscopy is the cornerstone of first-line management for the diagnosis and treatment of esophageal varices. However, there is no widely accepted standard strategy for esophagojejunal varices. We report a patient with esophagojejunal varices rupture 3 months after proximal gastrectomy treated with percutaneous transhepatic obliteration.

**Case presentation:**

A 50-year-old man who had undergone proximal gastrectomy with double-tract reconstruction for esophagogastric junctional cancer 3 months before was admitted to the hospital due to gastrointestinal perforation. We performed emergency surgery and abdominal symptoms and inflammatory response improved postoperative. However, on POD3, he had eruptive bleeding at the just anal side of esophagojejunal anastomosis. Endoscopic clipping was unsuccessful because the mucosa was fragile and easily lacerated. Contrast-enhanced CT scan revealed the dilatation of the jejunal vein flowing into the ascending jejunal limb. Therefore, he was diagnosed as esophagojejunal varices rupture and percutaneous transhepatic obliteration (PTO) was tried for hemostasis. The portal and superior mesenteric veins were catheterized with the percutaneous transhepatic approach. Contrast agent injection into the jejunal branch demonstrated retrograde flow to the azygos vein through esophagojejunal varices. The microcatheter was inserted into the variceal blood supply branch and 10 mL of 5% ethanolamine oleate with iopamidol was injected. After obliteration therapy, the superior mesenteric venogram showed complete occlusion of the variceal supply branch. The patient was discharged from the hospital without any complications after 14 days.

**Conclusion:**

PTO can be effective for gastroesophageal varices rupture with a dilated jejunal vein of the ascending limb, few supplying vessels, and little ascites.

## Introduction

Esophageal varices are one of the major complications of portal hypertension, and upper gastrointestinal endoscopy is the golden standard for the detection and treatment. However, the varices after proximal or total gastrectomy are uncommon because of the hemodynamics in which vessels serving as blood supply are all divided. Therefore, there is no widely accepted standard strategy for treatment. Herein, we report a patient with esophagojejunal varices rupture 3 months after proximal gastrectomy treated with percutaneous transhepatic obliteration (PTO).

## Case presentation

A 50-year-old man with loss of appetite and abdominal pain came to our hospital. His medical history included alcoholic liver cirrhosis (Child-Pugh score was B), and he had undergone proximal gastrectomy with double-tract reconstruction for esophagogastric junctional cancer 3 months before. On admission, he complained of severe abdominal distension and rebound tenderness. Laboratory examination showed white blood cells of 2800/μL; hemoglobin, 9.5 g/dL; platelet count, 180,000/μL; total-bilirubin, 2.5 mg/dL; and C-reactive protein, 0.93 mg/dL. Contrast-enhanced CT scan revealed free air with edematous wall thickening around of gastro-jejunal anastomosis. Gastrointestinal perforation was suspected and emergency surgery was performed. Intraoperatively, much ascites was stored in the peritoneal cavity, and 5 mm perforation was observed at gastro-jejunal anastomosis. There was no additional perforation (e.g., esophagojejunal anastomosis or jejunal-jejunal anastomosis), and no findings could be detected suggesting exacerbation of portal hypertension (e.g., dilation of the mesenteric vein, new formation of collateral circulation, or enlargement of splenomegaly). We performed suture closure, omentum covering, and wash drainage.

**Fig. 1:**
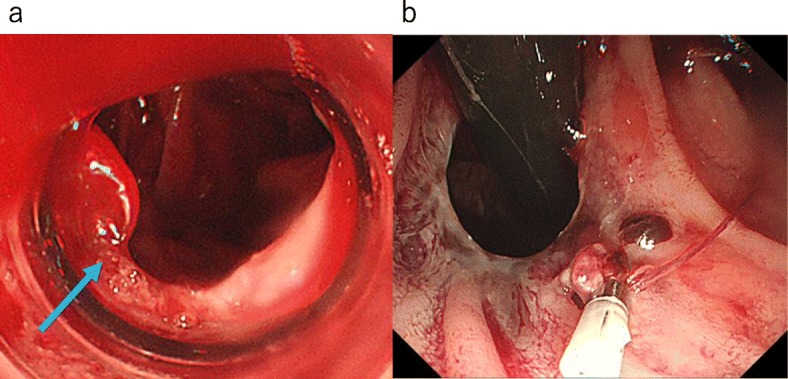
Emergency upper gastrointestinal endoscopy. **a** Exposed blood vessel with eruptive bleeding at the just anal side of esophagojejunum anastomosis (arrow). **b** Hemostasis was insufficient due to mucosal laceration

Abdominal symptoms and inflammatory response improved postoperative. However, on POD 3, he complained a large amount of tarry stool. Laboratory examination showed rapid progression of anemia and increased blood urea nitrogen (BUN)/creatinine ratio; hemoglobin, 6.8 g/dL; platelet count, 320,000/μL; asparate aminotransferase (AST), 136 U/L; alanine aminotransferase (ALT), 55 U/L; total bilirubin, 2.2 mg/dL; BUN, 20 mg/dL; and creatinine, 0.50 mg/dL. Emergency upper gastrointestinal endoscopy revealed an exposed blood vessel with eruptive bleeding at the just anal side of esophago-jejunum anastomosis (Fig. 1a). We attempted endoscopic clipping. However, the procedure was unsuccessful because the mucosa was fragile and easily lacerated. In the meanwhile, the patient became severe hemorrhagic shock and hemostasis was obtained (Fig. 1b).

**Fig. 2:**
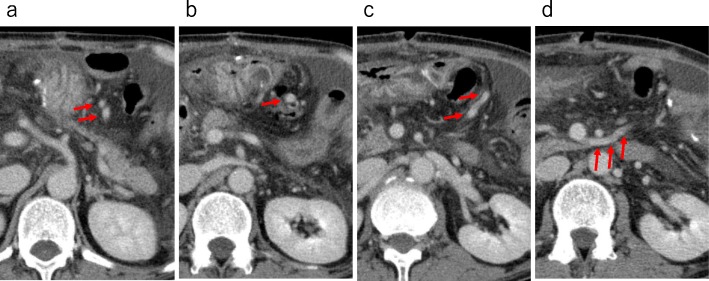
Contrast-enhanced CT scan. **a–d** Dilatation the branches of jejunal vein of the ascending jejunal limb (arrow)

**Fig. 3:**
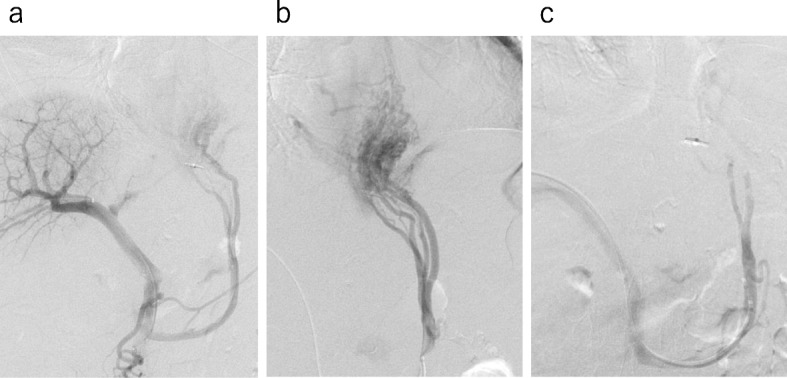
PTO. **a** The portal and superior mesenteric veins were catheterized with the percutaneous transhepatic approach. **b** Contrast agent injection into the jejunal branch demonstrated retrograde flow to the azygos vein through esophagojejunal varices. **c** After injection of EOI, the superior mesenteric venogram showed complete occlusion of the variceal supply branch

On POD 20, a contrast-enhanced CT scan was taken again because anemia continued to progress gradually despite fasting or RBC transfusion. The results revealed the dilatation of the jejunal vein flowing into the ascending jejunal limb (Fig. 2). Based on this finding, the patient was diagnosed with esophagojejunal varices rupture, and we tried PTO for hemostasis. The portal and superior mesenteric veins were catheterized with the percutaneous transhepatic approach. Contrast agent injection into the jejunal branch demonstrated retrograde flow to the azygos vein through esophagojejunal varices (Fig. 3a, b). The microcatheter was inserted into the variceal blood supply branch, and 50% glucose solution was injected to reduce blood flow. Then, we injected 10 mL of 5% ethanolamine oleate with iopamidol (5% EOI). After obliteration therapy, the superior mesenteric venogram showed complete occlusion of the variceal supply branch (Fig. 3c).

There were no complications during or after the operation, and the subsequent course showed no further bleeding. The patient discharged 14 days after the operation.

## Discussion

Variceal bleeding is a major complication in patients with portal hypertension and mostly occurred in the gastroesophageal area [1]. Ectopic varices are complex pressurized portosystemic venous collaterals occurring within the abdomen excluding the gastroesophageal area, and the incidence of ectopic varices is reported to be only 1–5% of all variceal bleed [2, 3]. A current survey of ectopic varices in Japan has been reported, and the most frequent sites of ectopic varices are the rectum in 44.5%, followed by the duodenum in 32.9% [4]. Varices in the esophagojejunal anastomotic area after proximal or total gastrectomy is uncommon because the supplying vessels such as left gastric vein, posterior gastric vein, or short gastric vein are all divided. Previous report showed the hemodynamics of these varices, which is supplied by branches of the jejunal vein of the arcade of the ascending jejunal limb and drained mainly through azygos [5]. Similar to our case, contrast-enhanced CT examination revealed that the dilated jejunal vein was configured as esophagojejunal varices [6]. Boku and colleagues suggested two mechanisms for these varices formations. First, the elimination of venous collaterals during the operation decreases available drainage veins that are decompressing a hypertensive portal system and may increase portal pressure. Second, ensuring hyperemia in the anastomotic area is expected to increase the venous outflow and thus raise venous pressure if drainage vessels are removed. In addition, neovascularization due to adhesion and inflammation in the peritoneal cavity is also considered to be the cause of varicose vein formation [7].

Previously reported promising therapies for ectopic varices include endoscopic injection sclerotherapy (EIS), endoscopic variceal ligation (EVL), surgery, balloon-occluded retrograde transvenous obliteration (BRTO), transjugular intrahepatic portosystemic shunt (TIPS), and PTO [8–10]. Emergent upper gastrointestinal endoscopy and EVL are the cornerstone of first-line management for the diagnosis and treatment of the esophageal varices [11]. However, this method is occasionally unsuccessful due to factors such as repeated insertions of the endoscope or a reduced field of view due to the attachment of the banding device which may fill with blood during active hemorrhage or the mucosa is fragile as in the present case [12, 13]. A surgical approach is also hard because of severe liver dysfunction and an intricate configuration of the alimentary tract after surgery. Therefore, we believe that PTO still has a role in the treatment of special types of varices. To our knowledge, there have been a few English case reports in the medical literature demonstrating the successful management of esophagojejunal varices treated with PTO after total gastrectomy. As for the proximal gastrectomy, this is the first case report [5, 7].

PTO is initially devised by Lunderquist in 1974 as an alternative to surgical intervention and occasionally used for acute variceal hemorrhage [14]. It enables understanding of hemodynamics relatively easily in angiography, as it is an antegrade embolization technique. In cases with multiple supplying vessels or a large amount of ascites, PTO is not the first choice because it may be ineffective unless all of the vessels are embolized and catheter manipulation is often extremely difficult through the ascites. Furthermore, the recurrence rate after PTO is higher than after endoscopic treatment because the original PTO technique using stainless-steel coils makes collateral circulation to the varices [15, 16]. However, the PTO technique is being developed with a microcatheter or microcoil [17]. We have applied this new technique in our case and have been able to superselectively obliterate the varices themselves as well as their feeding vein. Embolization was performed by 50% glucose solution and 5% ethanolamine oleate with iopamidol (5% EOI). When glucose and EOI pools in the variceal veins, they infiltrate and destroy the cell membrane of venous endothelial cells. Damage to the venous endothelium causes thrombus formation in varices [18]. This EOI mixture has reported to be effective and has good long-term results. Percutaneous transhepatic sclerotherapy (PTS) has also been recommended as a good option. In this technique, metallic coils are placed in the afferent veins to reduce the blood flow into the varices, after which a sclerosing agent is injected in the antegrade direction [19].

## Conclusion

We report a case of esophagojejunal varices rupture after proximal gastrectomy treated with PTO. This case suggests the effectiveness of PTO for patients with dilated jejunal vein of the ascending limb, few supplying vessels, and little ascites.

## Data Availability

The data that support the findings of this study are available from the corresponding author, N.S, upon reasonable request.

## Abbreviations

5% EOI: 5% ethanolamine oleate with iopamidol
ALT: Alanine aminotransferase
AST: Asparate aminotransferase
BRTO: Balloon occluded retrograde transvenous obliteration
BUN: Blood urea nitrogen
EIS: Endoscopic injection sclerotherapy
EVL: Endoscopic variceal ligation
PTO: Percutaneous transhepatic obliteration
PTS: Percutaneous transhepatic sclerotherapy
TIPS: Transjugular intrahepatic portosystemic shunt
